# 4-{[(1*S*,2*R*)-2-Hy­droxy­indan-1-yl]amino}­pent-3-en-2-one

**DOI:** 10.1107/S1600536812031169

**Published:** 2012-07-14

**Authors:** Ka Hyun Park, Min Jeong Go, Hwi Hyun Lee, Sungae Kim, Junseong Lee

**Affiliations:** a Department of Chemistry, Chonnam National University, Gwangju 500-757, Republic of Korea

## Abstract

In the mol­ecule of the title compound, C_14_H_17_NO_2_, the dihedral angle formed by the mean planes through the indan ring system and the amino­pentenone fragment is 83.26 (13)°. An intra­molecular N—H⋯O hydrogen bond is observed. In the crystal, mol­ecules are linked into one-dimensional chains extending along the [010] direction *via* O—H⋯O and C—H⋯O hydrogen bonds.

## Related literature

For metal complexes containing amino­indanol ligands, see: Lee *et al.* (2007 ▶); Flores-Lopes *et al.* (2000 ▶). For metal comlexes with acetyl­acetonate-type ligands, see: Patra *et al.* (2004 ▶); Jackson *et al.* (2006 ▶); Young *et al.* (2011 ▶).
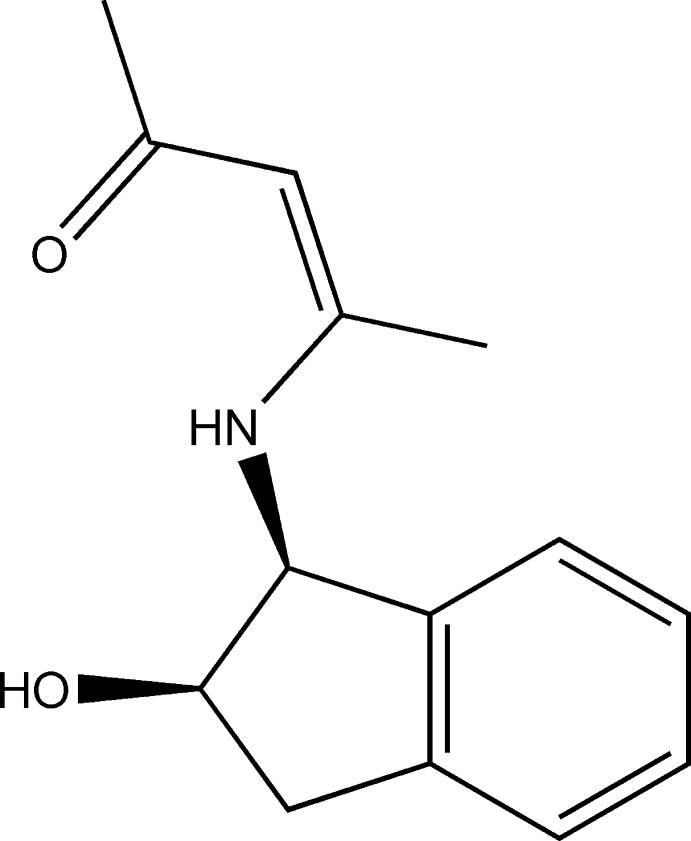


## Experimental

### 

#### Crystal data

C_14_H_17_NO_2_
*M* *_r_* = 231.29
Orthorhombic, 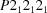

*a* = 8.3472 (5) Å
*b* = 11.2211 (7) Å
*c* = 13.4104 (9) Å
*V* = 1256.08 (14) Å^3^
*Z* = 4
Mo *K*α radiation
μ = 0.08 mm^−1^
*T* = 296 K
0.15 × 0.12 × 0.10 mm

#### Data collection

Bruker APEXII CCD diffractometer
Absorption correction: multi-scan (*SADABS*; Bruker, 2009 ▶) *T*_min_ = 0.988, *T*_max_ = 0.992
18304 measured reflections
1551 independent reflections
1198 reflections with *I* > 2σ(*I*)
*R*_int_ = 0.055

#### Refinement

*R*[*F* ^2^ > 2σ(*F* ^2^)] = 0.039
*wR*(*F* ^2^) = 0.095
*S* = 1.07
1551 reflections
164 parameters
H atoms treated by a mixture of independent and constrained refinement
Δρ_max_ = 0.12 e Å^−3^
Δρ_min_ = −0.12 e Å^−3^

### 

Data collection: *APEX2* (Bruker, 2009 ▶); cell refinement: *SAINT* (Bruker, 2009 ▶); data reduction: *SAINT*; program(s) used to solve structure: *SHELXS97* (Sheldrick, 2008 ▶); program(s) used to refine structure: *SHELXL97* (Sheldrick, 2008 ▶); molecular graphics: *SHELXTL* (Sheldrick, 2008 ▶); software used to prepare material for publication: *SHELXTL*.

## Supplementary Material

Crystal structure: contains datablock(s) I, global. DOI: 10.1107/S1600536812031169/rz2785sup1.cif

Supplementary material file. DOI: 10.1107/S1600536812031169/rz2785Isup2.cdx

Structure factors: contains datablock(s) I. DOI: 10.1107/S1600536812031169/rz2785Isup3.hkl

Supplementary material file. DOI: 10.1107/S1600536812031169/rz2785Isup4.cml

Additional supplementary materials:  crystallographic information; 3D view; checkCIF report

## Figures and Tables

**Table 1:** Hydrogen-bond geometry (Å, °)

*D*—H⋯*A*	*D*—H	H⋯*A*	*D*⋯*A*	*D*—H⋯*A*
O1—H101⋯O2^i^	0.80 (3)	2.03 (3)	2.829 (2)	179 (4)
N1—H201⋯O2	0.88 (2)	2.08 (2)	2.764 (3)	134 (2)
C9—H9*A*⋯O2^i^	0.97	2.44	3.254 (3)	141

Symmetry code: (i) 
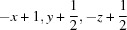
.

**Fig. 1.**
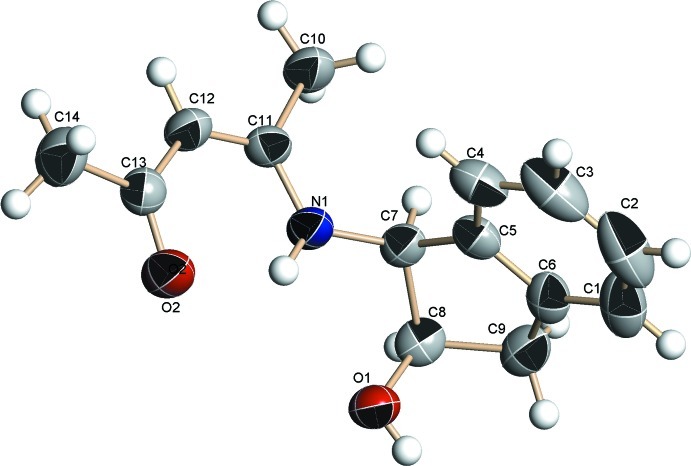
The molecular structure of the title compound, with displacement ellipsoids drawn at the 50% probability level. H atoms are shown as spheres of arbitrary radius.

**Fig. 2.**
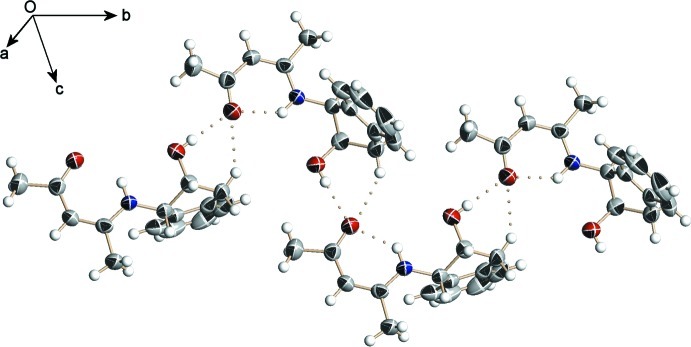
The one-dimensional chain structure extending along [010], with hydrogen bonds shown dotted lines. Displacement ellipsoids are drawn at the 50% probability level.

## supplementary crystallographic information

### Comment

Acetylacetonate derivatives have been widely used as chelating ligand systems (Patra *et al.*, 2004; Jackson *et al.*, 2006; Young *et al.*, 2011). Aminoindanol-type ligands have been also extensively used as chiral chelating ligands (Lee *et al.*, 2007; Flores-Lopes *et al.*, 2000). As part of our ongoing project on the synthesis of new O_2_N-type tridentate dianionic chelating ligand, the title compound was synthesized by the reaction of 2,4-pentanedione with (1*S*,2*R*)-(-)-*cis*-amino-2-indanol and 2,4-pentanedione, and its crystal structure is reported herein.

In the title compound (Fig. 1), the C8 carbon atom is displaced by 0.594 (2) Å from the mean plane defined by the C1–C7/C9 atoms of the indane ring system. The dihedral angle formed by the mean planes through the indane ring system and the approximately planar aminopentenone fragment [maximum deviation 0.063 (3) Å for atom C14] is 86.23 (13)°. The molecular conformation is stabilized by an intramolecular N—H···O hydrogen bond (Table 1). In the crystal structure (Fig. 2), molecules interact *via* intermolecular O—H···O and C—H···O hydrogen bonds to generate one-dimensional chains extending along the [0 1 0] direction.

### Experimental

A mixture of (1*S*,2*R*)-(-)-amino-2-indanol(0.149 g,1 mmol) and 2,4-pentanedione(0.100 g,1 mmol) was stirred in ethanol for 24 h in the presence of catalytic amount of *p*-toluene sulfonic acid. The residue, obtained by removing the solvent under vacuum, was recrystallized in hexane. The desired product was isolated as white crystals after the solution remained at -20 °C in a refrigerator for a few days (yield 80%, 0.183 g).

### Refinement

In the absence of significant anomalous scattering effects, 1121 Friedel pairs were merged in the last cycles of refinement. The absolute configuration was assigned on the basis of the known configuration of the indanyl alcohol employed in the synthesis. The C-bound H-atoms were included in calculated positions and treated as riding atoms, with C—H = 0.93-0.97 Å, and with *U*_iso_(H) = 1.2 *U*_eq_(C) or 1.5 *U*_eq_(C) for methyl H atoms. The amine and hydroxy H atoms were located in a difference Fourier map and refined isotropically.

### Crystal data

**Table:**

C_14_H_17_NO_2_	*Z* = 4
*M**_r_* = 231.29	*F*(000) = 496
Orthorhombic, *P*2_1_2_1_2_1_	*D*_x_ = 1.223 Mg m^−^^3^
Hall symbol: P 2ac 2ab	Mo *K*α radiation, λ = 0.71073 Å
*a* = 8.3472 (5) Å	µ = 0.08 mm^−^^1^
*b* = 11.2211 (7) Å	*T* = 296 K
*c* = 13.4104 (9) Å	Block, white
*V* = 1256.08 (14) Å^3^	0.15 × 0.12 × 0.10 mm

### Data collection

**Table:**

Bruker APEXII CCD diffractometer	1551 independent reflections
Radiation source: fine-focus sealed tube	1198 reflections with *I* > 2σ(*I*)
Graphite monochromator	*R*_int_ = 0.055
φ and ω scans	θ_max_ = 26.8°, θ_min_ = 2.4°
Absorption correction: multi-scan (*SADABS*; Bruker, 2009)	*h* = −10→10
*T*_min_ = 0.988, *T*_max_ = 0.992	*k* = −14→14
18304 measured reflections	*l* = −16→16

### Refinement

**Table:**

Refinement on *F*^2^	Primary atom site location: structure-invariant direct methods
Least-squares matrix: full	Secondary atom site location: difference Fourier map
*R*[*F*^2^ > 2σ(*F*^2^)] = 0.039	Hydrogen site location: inferred from neighbouring sites
*wR*(*F*^2^) = 0.095	H atoms treated by a mixture of independent and constrained refinement
*S* = 1.07	*w* = 1/[σ^2^(*F*_o_^2^) + (0.0432*P*)^2^ + 0.1264*P*] where *P* = (*F*_o_^2^ + 2*F*_c_^2^)/3
1551 reflections	(Δ/σ)_max_ < 0.001
164 parameters	Δρ_max_ = 0.12 e Å^−^^3^
0 restraints	Δρ_min_ = −0.12 e Å^−^^3^

### Special details

**Table:**

Geometry. All e.s.d.'s (except the e.s.d. in the dihedral angle between two l.s. planes) are estimated using the full covariance matrix. The cell e.s.d.'s are taken into account individually in the estimation of e.s.d.'s in distances, angles and torsion angles; correlations between e.s.d.'s in cell parameters are only used when they are defined by crystal symmetry. An approximate (isotropic) treatment of cell e.s.d.'s is used for estimating e.s.d.'s involving l.s. planes.
Refinement. Refinement of *F*^2^ against ALL reflections. The weighted *R*-factor *wR* and goodness of fit *S* are based on *F*^2^, conventional *R*-factors *R* are based on *F*, with *F* set to zero for negative *F*^2^. The threshold expression of *F*^2^ > σ(*F*^2^) is used only for calculating *R*-factors(gt) *etc*. and is not relevant to the choice of reflections for refinement. *R*-factors based on *F*^2^ are statistically about twice as large as those based on *F*, and *R*- factors based on ALL data will be even larger.

### Fractional atomic coordinates and isotropic or equivalent isotropic displacement parameters (Å^2^)

**Table:**

	*x*	*y*	*z*	*U*_iso_*/*U*_eq_	
N1	0.3729 (3)	0.46783 (17)	0.05082 (14)	0.0483 (5)	
H201	0.397 (3)	0.429 (2)	0.1058 (17)	0.051 (7)*	
O1	0.5423 (2)	0.58830 (16)	0.19665 (13)	0.0554 (5)	
H101	0.564 (4)	0.634 (3)	0.240 (2)	0.073 (10)*	
O2	0.3822 (2)	0.25196 (16)	0.14989 (12)	0.0616 (5)	
C1	0.1505 (4)	0.8140 (3)	0.2132 (2)	0.0750 (9)	
H1	0.1775	0.8852	0.2445	0.090*	
C2	−0.0019 (5)	0.7674 (4)	0.2230 (2)	0.0906 (12)	
H2	−0.0781	0.8080	0.2606	0.109*	
C3	−0.0421 (4)	0.6613 (4)	0.1774 (2)	0.0845 (10)	
H3	−0.1451	0.6311	0.1848	0.101*	
C4	0.0692 (3)	0.5991 (3)	0.1208 (2)	0.0629 (7)	
H4	0.0420	0.5274	0.0904	0.075*	
C5	0.2214 (3)	0.6462 (2)	0.11056 (16)	0.0476 (6)	
C6	0.2630 (3)	0.7535 (2)	0.15616 (16)	0.0510 (6)	
C7	0.3656 (3)	0.5968 (2)	0.05584 (16)	0.0452 (5)	
H7	0.3632	0.6275	−0.0126	0.054*	
C8	0.5060 (3)	0.6566 (2)	0.11036 (17)	0.0505 (6)	
H8	0.5997	0.6638	0.0667	0.061*	
C9	0.4372 (3)	0.7797 (2)	0.13634 (19)	0.0582 (7)	
H9A	0.4889	0.8127	0.1949	0.070*	
H9B	0.4495	0.8348	0.0812	0.070*	
C10	0.2653 (4)	0.4617 (2)	−0.11916 (16)	0.0625 (7)	
H10A	0.1859	0.5216	−0.1060	0.094*	
H10B	0.2224	0.4038	−0.1646	0.094*	
H10C	0.3583	0.4982	−0.1481	0.094*	
C11	0.3113 (3)	0.4010 (2)	−0.02268 (15)	0.0464 (6)	
C12	0.2908 (3)	0.2795 (2)	−0.01416 (18)	0.0533 (6)	
H12	0.2533	0.2394	−0.0702	0.064*	
C13	0.3212 (3)	0.2108 (2)	0.07099 (19)	0.0508 (6)	
C14	0.2757 (4)	0.0807 (2)	0.0684 (2)	0.0757 (9)	
H14A	0.3551	0.0349	0.1033	0.113*	
H14B	0.2697	0.0543	0.0005	0.113*	
H14C	0.1735	0.0701	0.1000	0.113*	

### Atomic displacement parameters (Å^2^)

**Table:**

	*U*^11^	*U*^22^	*U*^33^	*U*^12^	*U*^13^	*U*^23^
N1	0.0661 (13)	0.0434 (11)	0.0353 (9)	−0.0011 (11)	−0.0078 (10)	0.0043 (9)
O1	0.0607 (11)	0.0556 (11)	0.0498 (10)	−0.0050 (9)	−0.0098 (9)	−0.0020 (9)
O2	0.0826 (12)	0.0542 (10)	0.0479 (9)	−0.0051 (10)	−0.0098 (10)	0.0080 (9)
C1	0.110 (3)	0.0578 (17)	0.0568 (16)	0.0261 (19)	0.0156 (18)	0.0162 (14)
C2	0.086 (3)	0.102 (3)	0.084 (2)	0.044 (2)	0.031 (2)	0.039 (2)
C3	0.0562 (18)	0.116 (3)	0.081 (2)	0.016 (2)	0.0068 (17)	0.041 (2)
C4	0.0527 (16)	0.0772 (18)	0.0587 (15)	0.0003 (15)	−0.0092 (13)	0.0167 (15)
C5	0.0506 (15)	0.0525 (14)	0.0398 (12)	0.0034 (12)	−0.0039 (11)	0.0123 (11)
C6	0.0691 (17)	0.0434 (13)	0.0406 (11)	0.0078 (13)	0.0052 (12)	0.0107 (11)
C7	0.0595 (14)	0.0417 (12)	0.0344 (10)	−0.0033 (12)	−0.0017 (11)	0.0053 (10)
C8	0.0526 (15)	0.0533 (14)	0.0455 (13)	−0.0084 (12)	0.0072 (11)	0.0022 (12)
C9	0.0829 (19)	0.0444 (14)	0.0473 (13)	−0.0088 (13)	−0.0013 (14)	0.0063 (11)
C10	0.086 (2)	0.0582 (15)	0.0431 (12)	0.0037 (16)	−0.0140 (14)	−0.0024 (12)
C11	0.0524 (14)	0.0533 (14)	0.0335 (11)	0.0031 (12)	−0.0023 (10)	−0.0016 (10)
C12	0.0666 (16)	0.0505 (15)	0.0428 (12)	−0.0006 (13)	−0.0089 (12)	−0.0066 (11)
C13	0.0530 (15)	0.0484 (14)	0.0510 (14)	0.0014 (12)	0.0005 (12)	−0.0005 (11)
C14	0.096 (2)	0.0504 (15)	0.0802 (19)	−0.0035 (16)	−0.0055 (18)	0.0013 (15)

### Geometric parameters (Å, º)

**Table:**

N1—C11	1.341 (3)	C7—C8	1.536 (3)
N1—C7	1.450 (3)	C7—H7	0.9800
N1—H201	0.88 (2)	C8—C9	1.536 (3)
O1—C8	1.421 (3)	C8—H8	0.9800
O1—H101	0.79 (3)	C9—H9A	0.9700
O2—C13	1.262 (3)	C9—H9B	0.9700
C1—C2	1.382 (5)	C10—C11	1.512 (3)
C1—C6	1.389 (4)	C10—H10A	0.9600
C1—H1	0.9300	C10—H10B	0.9600
C2—C3	1.380 (5)	C10—H10C	0.9600
C2—H2	0.9300	C11—C12	1.379 (3)
C3—C4	1.388 (4)	C12—C13	1.401 (3)
C3—H3	0.9300	C12—H12	0.9300
C4—C5	1.383 (4)	C13—C14	1.509 (3)
C4—H4	0.9300	C14—H14A	0.9600
C5—C6	1.395 (4)	C14—H14B	0.9600
C5—C7	1.515 (3)	C14—H14C	0.9600
C6—C9	1.507 (4)		
			
C11—N1—C7	125.2 (2)	O1—C8—H8	111.1
C11—N1—H201	115.0 (15)	C7—C8—H8	111.1
C7—N1—H201	118.0 (15)	C9—C8—H8	111.1
C8—O1—H101	107 (2)	C6—C9—C8	103.0 (2)
C2—C1—C6	119.3 (3)	C6—C9—H9A	111.2
C2—C1—H1	120.3	C8—C9—H9A	111.2
C6—C1—H1	120.3	C6—C9—H9B	111.2
C3—C2—C1	120.5 (3)	C8—C9—H9B	111.2
C3—C2—H2	119.7	H9A—C9—H9B	109.1
C1—C2—H2	119.7	C11—C10—H10A	109.5
C2—C3—C4	120.9 (3)	C11—C10—H10B	109.5
C2—C3—H3	119.5	H10A—C10—H10B	109.5
C4—C3—H3	119.5	C11—C10—H10C	109.5
C5—C4—C3	118.5 (3)	H10A—C10—H10C	109.5
C5—C4—H4	120.8	H10B—C10—H10C	109.5
C3—C4—H4	120.8	N1—C11—C12	122.7 (2)
C4—C5—C6	121.0 (2)	N1—C11—C10	118.3 (2)
C4—C5—C7	129.7 (2)	C12—C11—C10	119.0 (2)
C6—C5—C7	109.3 (2)	C11—C12—C13	126.1 (2)
C1—C6—C5	119.7 (3)	C11—C12—H12	117.0
C1—C6—C9	130.8 (3)	C13—C12—H12	117.0
C5—C6—C9	109.4 (2)	O2—C13—C12	123.7 (2)
N1—C7—C5	114.9 (2)	O2—C13—C14	118.3 (2)
N1—C7—C8	115.2 (2)	C12—C13—C14	117.9 (2)
C5—C7—C8	102.48 (17)	C13—C14—H14A	109.5
N1—C7—H7	107.9	C13—C14—H14B	109.5
C5—C7—H7	107.9	H14A—C14—H14B	109.5
C8—C7—H7	107.9	C13—C14—H14C	109.5
O1—C8—C7	108.34 (18)	H14A—C14—H14C	109.5
O1—C8—C9	112.34 (19)	H14B—C14—H14C	109.5
C7—C8—C9	102.4 (2)		

### Hydrogen-bond geometry (Å, º)

**Table:**

*D*—H···*A*	*D*—H	H···*A*	*D*···*A*	*D*—H···*A*
O1—H101···O2^i^	0.80 (3)	2.03 (3)	2.829 (2)	179 (4)
N1—H201···O2	0.88 (2)	2.08 (2)	2.764 (3)	134 (2)
C9—H9*A*···O2^i^	0.97	2.44	3.254 (3)	141

Symmetry code: (i) −*x*+1, *y*+1/2, −*z*+1/2.
